# Use of a Low-Cost Portable 3D Virtual Reality Simulator for Psychomotor Skill Training in Minimally Invasive Surgery: Task Metrics and Score Validity

**DOI:** 10.2196/19723

**Published:** 2020-10-27

**Authors:** Fernando Alvarez-Lopez, Marcelo Fabián Maina, Fernando Arango, Francesc Saigí-Rubió

**Affiliations:** 1 Faculty of Health Sciences Universidad de Manizales Manizales Colombia; 2 Faculty of Health Sciences Universitat Oberta de Catalunya Barcelona Spain; 3 Faculty of Psychology and Education Sciences Universitat Oberta de Catalunya Barcelona Spain

**Keywords:** simulation training, minimally invasive surgical procedures, medical education, user-computer interface, computer-assisted surgery, Leap Motion Controller

## Abstract

**Background:**

The high cost and low availability of virtual reality simulators in surgical specialty training programs in low- and middle-income countries make it necessary to develop and obtain sources of validity for new models of low-cost portable simulators that enable ubiquitous learning of psychomotor skills in minimally invasive surgery.

**Objective:**

The aim of this study was to obtain validity evidence for relationships to other variables, internal structure, and consequences of testing for the task scores of a new low-cost portable simulator mediated by gestures for learning basic psychomotor skills in minimally invasive surgery. This new simulator is called SIMISGEST-VR (Simulator of Minimally Invasive Surgery mediated by Gestures - Virtual Reality).

**Methods:**

In this prospective observational validity study, the authors looked for multiple sources of evidence (known group construct validity, prior videogaming experience, internal structure, test-retest reliability, and consequences of testing) for the proposed SIMISGEST-VR tasks. Undergraduate students (n=100, reference group), surgical residents (n=20), and experts in minimally invasive surgery (n=28) took part in the study. After answering a demographic questionnaire and watching a video of the tasks to be performed, they individually repeated each task 10 times with each hand. The simulator provided concurrent, immediate, and terminal feedback and obtained the task metrics (time and score). From the reference group, 29 undergraduate students were randomly selected to perform the tasks 6 months later in order to determine test-retest reliability.

**Results:**

Evidence from multiple sources, including strong intrarater reliability and internal consistency, considerable evidence for the hypothesized consequences of testing, and partial confirmation for relations to other variables, supports the validity of the scores and the metrics used to train and teach basic psychomotor skills for minimally invasive surgery via a new low-cost portable simulator that utilizes interaction technology mediated by gestures.

**Conclusions:**

The results obtained provided multiple sources of evidence to validate SIMISGEST-VR tasks aimed at training novices with no prior experience and enabling them to learn basic psychomotor skills for minimally invasive surgery.

## Introduction

### Background

The advent of minimally invasive surgery in the mid-1980s [1] led to an increase in the number of iatrogenic bile duct injuries when many surgeons worldwide switched from open surgery to minimally invasive surgery without any prior training [2-8]. As a result, simulation has since become a valuable tool for learning motor skills for minimally invasive surgery. Many studies have demonstrated that simulation is a useful tool for learning motor skills for minimally invasive surgery and that learned skills can be transferred to the operating theatre [9-19].

The first virtual reality (VR) simulator for minimally invasive surgery training was MIST-VR (Minimally Invasive Surgery Training - Virtual Reality) [20]. Evidence for construct validity was established in 1998 [21], and evidence for predictive validity was obtained in 2002 [9,22]. Subsequently, evidence for concurrent validity was also demonstrated [23-25].

Recent years have seen the development of low-cost gesture-based touchless devices that can interact with 3D virtual environments, among them Kinect (Microsoft Corp), the Leap Motion Controller (Leap Motion Inc), and the Myo armband (Thalmic Labs) [26].

The Leap Motion Controller was launched in May 2012. It is based on the principle of infrared optical tracking, which detects the positions of fine objects such as fingertips or pen tips in a Cartesian plane; its interaction zone is an inverted cone of approximately 0.23 m^3^, and it has a motion detection range between 20 mm and 600 mm [27,28]. It measures 76 mm × 30 mm × 13 mm and weighs 45 g. It has 3 infrared emitters and 2 infrared cameras that capture the movements generated within the interaction zone [29,30]. The manufacturer reports an accuracy of 0.01 mm for fingertip detection, although one independent study showed an accuracy of 0.7 mm [31]. Although the Leap Motion Controller is designed mainly to detect hand motions, it can track objects such as pencils and laparoscopic surgical forceps [32-34].

The Leap Motion Controller has been used as a tool for the manipulation of medical images in the fields of interventional radiology and image-guided surgery or when there is a risk of contamination through contact (autopsy rooms, for example), for touchless control (operating theatre lights and tables) and for simulation (minimally invasive surgery and robotic surgery). Various authors have used the Leap Motion Controller to develop simulators that track hand or instrument movements [26,32-39]. A paper by Lahanas [35] describes using Leap Motion Controller to simulate 3 tasks: camera navigation, instrument navigation, and bimanual operation; 28 expert surgeons and 21 reference individuals took part in the study. The experts significantly outperformed novices in all assessment metrics for instrument navigation and bimanual operation.

Simulators for learning skills for minimally invasive surgery can be classified into 3 types: traditional box trainers, augmented reality simulators (hybrid), and VR simulators [40,41]. Simulation has become a valuable tool for learning basic motor skills in surgery, but access to simulators remains problematic, especially in low- and middle-income countries, because of their high cost. Consequently, that makes it necessary to develop and validate the metrics and scores of low-cost portable simulators [42-44].

The aim of this study was to evaluate a simulation instrument, SIMISGEST-VR (Simulator of Minimally Invasive Surgery mediated by Gestures - Virtual Reality), and to document the sources of validity evidence for task scores, relations to other variables, internal structure, consequences of testing, and response process.

### Hypotheses

To that end, 3 hypotheses were formulated:

#### Hypothesis 1: Validity Evidence for Relations to Other Variables

The first hypothesis aims to demonstrate that the test scores discriminate between a reference group (no prior experience), surgical residents (less experienced), and surgeons (more experienced), showing that the experts already have the basic psychomotor skills being measured, and similarly, that videogaming experience is correlated with better performance in simulator tasks, regardless of the level of training and experience.

#### Hypothesis 2: Evidence for Internal Structure

The intrarater test-retest assumes that, if a reference individual is not exposed to simulators in the period of time between the 2 complete simulator exercises, there will be no significant differences in performance between the first and second exercises.

#### Hypothesis 3: Evidence for Consequences of Testing

Regarding evidence for consequences of testing, the reference group learning will be demonstrated by improvements in the metrics and the final score when comparing the first and the tenth attempt in each task.

## Methods

### Study Design

This was a prospective observational validity study. The current unified standard considers that all validity is construct validity and, as such, requires evidence from 5 sources [45-52].

*Content evidence* includes a description of the steps taken to ensure that test content reflects, in a relevant way, the construct or characteristic being measured. The results obtained from the survey assessing fidelity to the criterion and content-related validity evidence for SIMISGEST-VR showed that all 30 participants felt that most aspects of the simulator were adequately realistic and that it could be used as a tool for teaching basic psychomotor skills in laparoscopic surgery (Likert score: range 4.07-4.73). The sources of content-related validity evidence showed that our simulator was a reliable training tool and that the tasks enabled learning of the basic psychomotor skills required in minimally invasive surgery (Likert score: range 4.28-4.67) [53].

Evidence for *relations to other variables* refers to the statistical association between the test scores and other characteristics or external measures that have theoretical relations, such as level of training, level of experience, prior videogaming experience, and scores for other already validated instruments. One of the most common correlations is *known group construct validity* (ie, the correlation between performance scores and level of training and experience) [54]. Relations may be positive (convergent or predictive) or negative (divergent or discriminant) depending on the constructs being measured [55]. This study explored the relations between performance scores and the level of training, experience, and prior videogaming experience.

Evidence for *internal structure* includes data that evaluate the relations between the individual items of the assessment, and how they correlate to the construct. It includes measures of reliability, reproducibility, and factor analysis. Reliability is a necessary but insufficient condition for validity [56]. Intrarater reliability was obtained using the test-retest method, which assesses the stability of responses over time [57]. Test-retest reliability was explored through blinded rerating after an interval of 6 months in the reference group. The randomly selected participants were asked whether they had had additional experience of using simulators during that period of time [56]. The answer was “no” in all cases. The data produced by this second test were not taken into account in the evidence for the construct validity study. Worster and Haines [58] noted that there was no published recommendation for the proportion of data that should be checked but that 10% was common. In this study, 29% of the reference individuals were included in the test-retest study. The demonstration of reliability is mandatory before an evaluation can be shown to be valid [54].

Evidence for *consequences* refers to the impact, benefit, or danger of assessment itself and the resulting decisions and actions. Yet, simply demonstrating consequences, even significant and impressive ones, does not constitute validity evidence unless investigators explicitly demonstrate that these consequences have an impact on score interpretation (validity) [46,55]. Evidence for consequences falls within a spectrum between high-stake examinations, licensing examinations, or low-stake examinations such a self-assessment used for formative feedback alone [54]. In our case, we hoped to obtain evidence to demonstrate that the reference group had managed to achieve the learning curve.

Evidence for *response process* includes theoretical and empirical analyses evaluating the extent to which the assessors’ and respondents’ responses are aligned to the construct. It includes an evaluation of safety, of quality control, and of the actors’ thoughts and actions during the assessment. The response process also includes the accuracy of data collection and entry into the database [54]. This type of evidence can be difficult to demonstrate because data are often qualitative [55].

### Participants and Simulator Test Methodology

Participating in this study were minimally invasive surgery expert surgeons (n=28) in a range of surgical specialties, each who had performed more than 100 procedures, surgical residents (n=20) in a range of surgical specialties from the University of Caldas (in Manizales, Colombia), each who had performed fewer than 50 procedures (basic training: n=15; advanced training: n=5), and medical undergraduate students (n=100) from the University of Caldas and the University of Manizales who had no experience performing minimally invasive surgical procedures. The expert surgeons worked in the following specialties: general surgery 8 (28.5%), pediatric surgery 5 (17.8%), neurosurgery 4 (14.2%), colorectal surgery 3 (10.7%), orthopedic surgery 3 (10.7%), gynecological surgery 2 (7.1%), urological surgery 1 (3.5%), thoracic surgery 1 (3.5%), and vascular surgery 1 (3.5%).

All participants completed a questionnaire providing demographic data (Multimedia Appendix 1) and information about the dominant hand, level of training, levels of minimally invasive surgery skills, prior training with simulators, and experience with videogaming or VR devices.

After the instructor had given basic instructions about using the simulator and had shown a video of each task to be performed, the study participants performed 10 repetitions of tasks 1, 2, 4, 5, and 6 with each hand. Task 3 was repeated 10 times because both hands were considered dominant. The instructor did not give additional feedback, but the simulator did provide concurrent feedback (visual and auditory feedback while performing each task), immediate feedback (displaying the results in terms of time, accuracy and errors at the end of each task), and terminal feedback (performance curve and final score). The participants were able to watch the demonstration videos again at any time. For the test-retest reliability study, 29 participants were randomly selected from the reference group. They repeated the entire exercise 6 months after the first exercise; none were exposed to any type of simulator during that period of time. One of the authors (FAL) supervised and photographically documented each exercise.

### SIMISGEST-VR

SIMISGEST-VR was developed using design-based research [59-63]. A previously published article [53] describes in detail the development of the device and a study assessing fidelity to the criterion and content-related validity evidence.

### Virtual Environment

The virtual environment consisted of the following modules: registration to collect users’ demographic data and a tutorial to show demonstration videos of the tasks to be performed.

**Figure 1:**
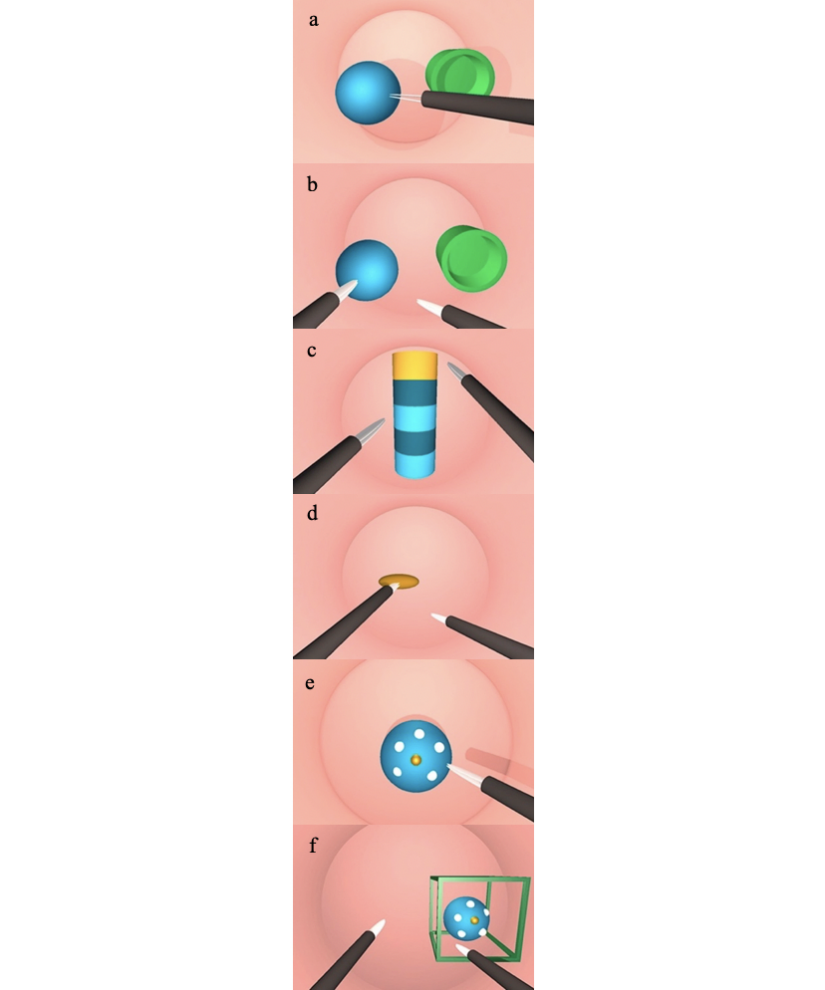
Task screenshots.

SIMISGEST-VR supports 6 tasks, each of which corresponds to a surgical equivalent (Table 1; Figure 1). The tasks were adapted from MIST-VR (Mentice Inc) [20,64,65]. MIST-VR is the simulator on which the highest number of validation studies have been conducted, and they have demonstrated, on multiple occasions, that the skills that are learned can be transferred to the operating theatre [9,21,66-73].

Except for task 3, all tasks had the option of configuring the dominant hand during the exercise; task 3 required the simultaneous use of both hands and therefore both played a dominant function. Given its level of difficulty, this task was performed last in all cases. The online virtual environment ran on Windows (Microsoft Inc) and MacOS (Apple Inc) platforms.

**Table 1:** Description of the tasks and their surgical equivalents. Adapted from Sutton et al [20].

Task number	Task name	Description	Surgical equivalent	Learning objective
Task 1	Grip and placement	Take the sphere with one hand and move it to a new location within the workspace	Gripping and retraction of tissue to a given position, placement of clips and hemostasis, use of extractor bags	Visual-spatial perception and eye-hand coordination
Task 2	Transfer and placement of an object	Take the sphere, transfer it to another instrument and place it inside a hollow cylinder	Transfer of a needle between a clamp and a needle holder	Visual-spatial perception, eye-hand coordination, and use of both hands in a complementary manner
Task 3	Cross	Instruments travel along a surface in a 3D cylinder	Small intestine exploration	Coordinated use of both the dominant and nondominant hands and ambidexterity
Task 4	Removal and reinsertion of instruments	Removal of instruments from the operative site and reinsertion	One instrument stabilizes one organ while the other is removed from the field and reintroduced	Visual-spatial perception, use of both hands in a complementary manner, and depth perception
Task 5	Diathermy	Cauterize a series of targets located in a fixed sphere	Cauterize a bleeding blood vessel	Visual-spatial perception, time of diathermy, and accuracy of movements
Task 6	Target manipulation and diathermy	Take the sphere with the instrument and place it inside a virtual space represented by a cube and cauterize a series of targets with the other hand.	Present and set a target to cauterize	Visual-spatial perception, time of diathermy, and accuracy of movements

### Metrics

The metrics were established using 5 parameters: time (velocity), efficiency of movement for the right and left hands [21,74], economy of diathermy, error and accuracy (penalty) [75,76], and final score.

### Feedback

Feedback is essential [77]. Training on a simulator should have 3 purposes: to improve performance, to make performance consistent, and to reduce the number of errors [78]. The haptic sensation and concurrent feedback were simulated using sound signals, color changes in the objects, and movement of the object when an undue collision occurred between the different components of the environment or when an error occurred during the task (concurrent feedback). For SIMISGEST-VR, we adopted 3 types of feedback: concurrent, which was provided while the task is being performed; immediate, which was provided at the end of each task when the system provides information on the presence or absence of errors, efficiency, and the time taken; and terminal, which was provided at the end of each training session when the system provides a series of graphs and tables that show performance over time [79-83].The data generated by the program were stored on an SQL (structured query language) database engine integrated into the simulation software.

### Hardware

**Figure 2:**
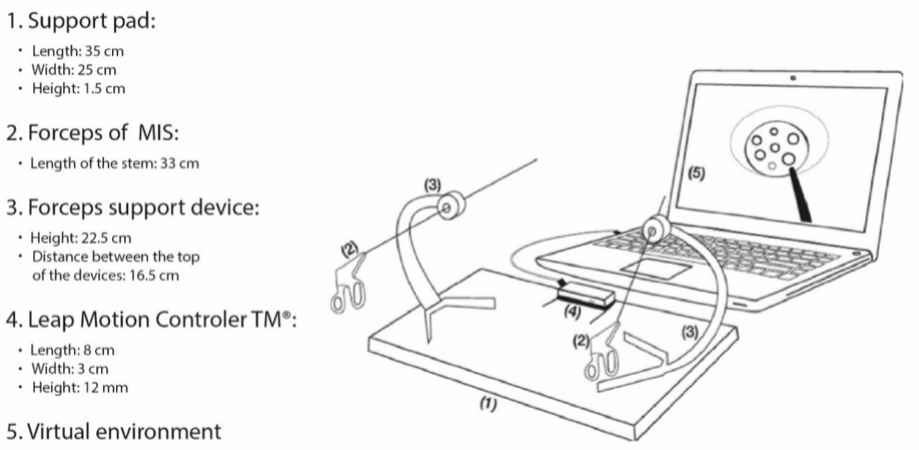
Diagram of the artifact. MIS: minimally invasive surgery.

Two laparoscopic forceps were used. In fact, we used simulated forceps made using 3D printers. These minimally invasive surgery forceps did not need to be functional. The final device with all its components assembled is shown in Figure 2. Figure 2 shows the fixing pad (1) for the Leap Motion Controller and the mounting support devices (3) for the minimally invasive surgery laparoscopic forceps (2), which allow simulation of the fulcrum effect; the Leap Motion Controller (4), responsible for detecting the movements of the instruments; and the computer, which, by means of the software programs running on it, administers the virtual environment and the metrics, and provides feedback and the final performance score on the screen (5) where the 3D virtual environment is displayed.

To perform the test, a 13-inch MacBook Pro (Apple Inc) was used, which served as a screen, ran the 3D virtual environment, and stored metrics data.

### Data Analysis

Continuous data are presented in a frequency distribution table by mean and standard deviation. The Shapiro-Wilk test was used to assess normality. Categorical data are also presented in a frequency distribution table. Since the metrics data were not normally distributed, nonparametric tests were used to assess the hypotheses. Regarding hypothesis 1, the differences in the scores and time taken to perform the first trial in each task between novices and experts were compared using the Wilcoxon signed-rank test. Among the novices, the final scores of the tenth trial in each task were compared by prior videogaming experience using the Kolmogorov-Smirnov test. To assess hypothesis 2, internal consistency was calculated using Cronbach α. In addition, test-retest reliability was assessed by comparing the tenth trial in each task performed initially and repeated 6 months later using the Spearman correlation coefficient. To assess hypothesis 3, the scores and time taken in the first and last trials in each task were compared by level of training using the Wilcoxon signed-rank test. In addition, excess diathermy in the first and last trials in tasks 5 and 6 was calculated by level of training using the Wilcoxon signed-rank test. *P*<.05 as level of statistical significance was established. Statistical analysis was performed using Stata (version 15.0; StataCorp LLC).

## Results

### Demographic Profile

Regarding prior experience with simulators, 35% (7/20) of the surgical residents and 36% (10/28) of the surgeons surveyed said they did not have any. Among the surgical residents, only 15% (3/20) had experience with VR simulators, and none had any experience with hybrid ones.

When videogaming experience was assessed, the low percentage of frequent gaming (daily or weekly) was striking: only 28% (28/100) in the reference group, 20% (4/20) among surgical residents, and 14% (4/28) among experts (Table 2).

**Table 2:** Demographic profile of study participants.

Variable		Reference group (n=100)	Surgical residents (n=20)	Surgeons (n=28)
**Gender, n (%)**				
	Female	47 (47)	10 (50)	4 (15)
	Male	53 (53)	10 (50)	24 (86)
Age		23.5 (0.28)	28.4 (0.54)	47 (2.12)
**Dominant hand, n (%)**				
	Right	89 (89)	19 (95)	27 (96)
	Left	11 (11)	1 (5)	1 (4)
**Experience with simulators, n (%)**				
	Yes	1 (1)	13 (65)	18 (64)
	No	99 (99)	7 (35)	10 (36)
**Type of simulator, n (%)**				
	Not applicable	99 (99)	7 (35)	10 (25)
	Virtual reality	1 (1)	3 (15)	7 (36)
	Physical	0 (0)	10 (50)	10 (25)
	Hybrid/augmented reality	0 (0)	0 (0)	1 (4)
**Videogaming experience, n (%)**				
	Yes	72 (72)	15 (75)	14 (50)
	No	28 (28)	5 (25)	14 (50)
**Videogaming frequency, n (%)**				
	Not applicable	26 (26)	4 (20)	14 (50)
	Daily	1 (1)	1 (5)	0 (0)
	Weekly	27 (27)	3 (15)	4 (14)
	Monthly	6 (6)	3 (15)	3 (11)
	Occasionally	40 (40)	9 (45)	7 (25)
**Minimally invasive surgery experience, n (%)**				
	None	37 (37)	3 (15)	0 (0)
	Basic camera manipulation	63 (63)	6 (30)	0 (0)
	Basic operator level	0 (0)	11 (55)	10 (36)
	Intermediate operator level	0 (0)	0 (0)	10 (36)
	Advanced operator level	0 (0)	0 (0)	8 (29)

### Validity Hypothesis 1: Relations to Other Variables

**Figure 3:**
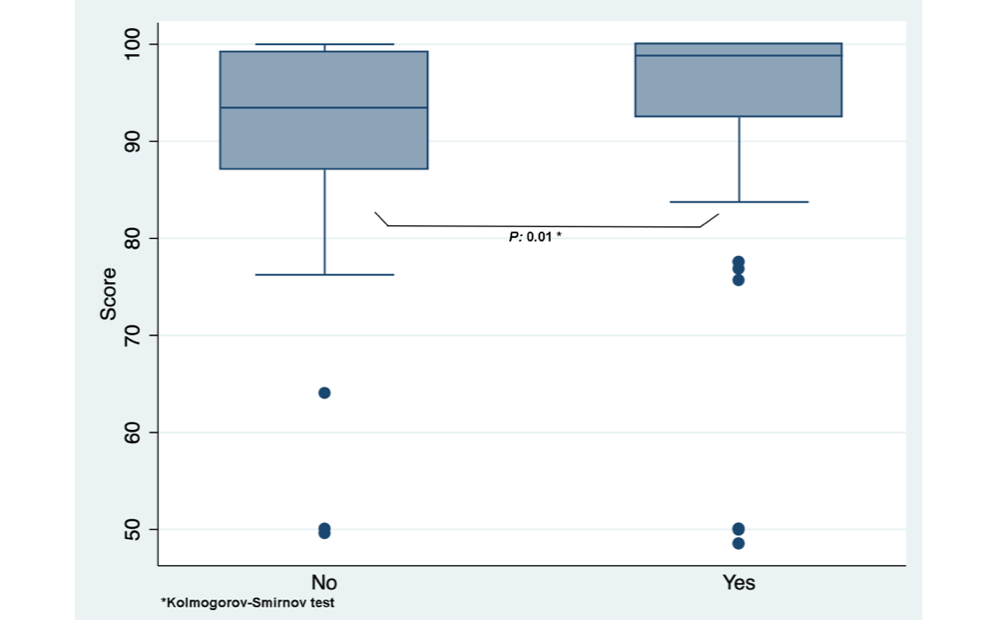
Box plot of scores in task 5 performed by the reference group, by prior videogaming experience.

**Figure 4:**
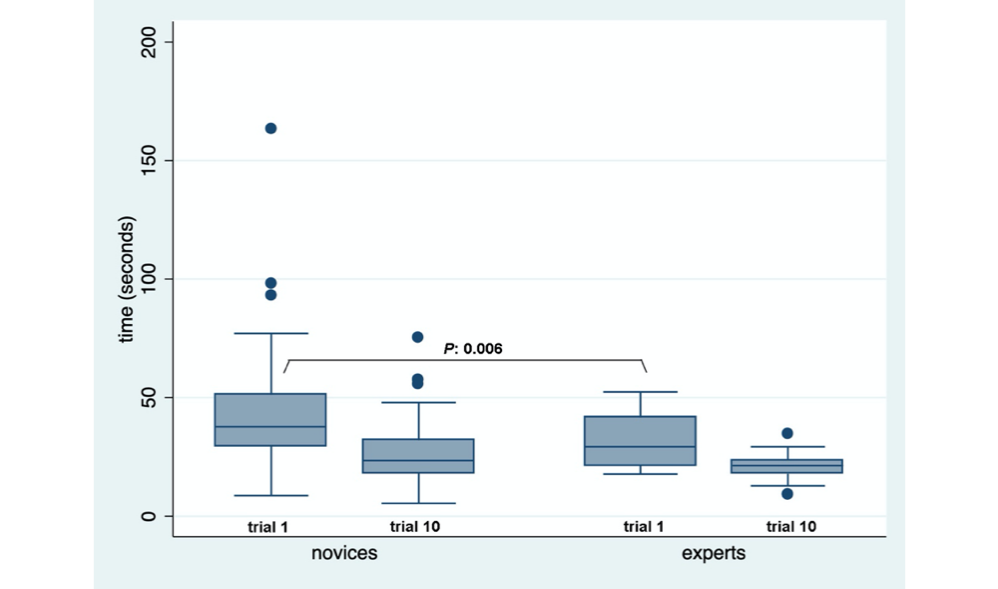
Box plot of times in task 3, by level of training.

To explore validity evidence for relations to other variables, we compared the SIMISGEST-VR test scores across experience levels (known group construct validity). No statistically significant differences were found in the scores of the first trial in each task between novices and experts; however, the times taken to perform tasks 3 (*P*=.006) and 6 (*P*=.02) were statistically significantly lower for experts compared to those of the reference group (Table 3). Performance in task 5 was better for novices who had prior videogaming experience (*P*=.01), as shown in Figure 3. When time was considered as a metric in task 3, a statistically significant difference (*P*=.006) was found between the reference group and the experts in performing the first trial (Figure 4).

**Table 3:** Trial 1 scores and time between novices and experts.

Metric and task		*P* value
**Score**		
	Task 1	.58
	Task 2	.13
	Task 3	.33
	Task 4	.18
	Task 5	.77
	Task 6	.27
**Time**		
	Task 1	.53
	Task 2	.34
	Task 3	.006
	Task 4	.26
	Task 5	.28
	Task 6	.02

### Validity Hypothesis 2: Internal Structure

The items demonstrated high internal consistency (Cronbach α=.81). Regarding the final scores in all the tasks, no statistically significant differences were found between the first exercises and those 6 months later for the randomly selected participants from the reference group (Table 4); when time was assessed as a metric, statistically significant differences were found for tasks 4 (trial 10: *P*=.048) and 6 (trial 10: *P*=.03). This demonstrates full evidence for the internal structure and test-retest reliability with respect to the score and partial evidence with respect to time as a metric (Table 4).

**Table 4:** Test-retest score for novices showing reliability between trials.

Metric, task, and trial comparison			Spearman correlation coefficient		*P* value	
**Score (initial vs 6 months later)**						
	**Task 1**					
		Trial 1		0.200		.23
		Trial 10		–0.294		.12
	**Task 2**					
		Trial 1		0.0846		.66
		Trial 10		–0.256		.18
	**Task 3**					
		Trial 1		–0.036		.85
		Trial 10		0.150		.44
	**Task 4**					
		Trial 1		0.120		.53
		Trial 10		0.338		.07
	**Task 5**					
		Trial 1		0.341		.07
		Trial 10		0.035		.85
	**Task 6**					
		Trial 1		–0.321		.09
		Trial 10		–0.030		.88
**Time (initial vs 6 months later)**						
	**Task 1**					
		Trial 1		–0.005		.98
		Trial 10		0.243		.20
	**Task 2**					
		Trial 1		–0.082		.67
		Trial 10		–0.216		.26
	**Task 3**					
		Trial 1		0.121		.53
		Trial 10		0.359		.06
	**Task 4**					
		Trial 1		–0.141		.46
		Trial 10		0.370		.048
	**Task 5**					
		Trial 1		0.271		.16
		Trial 10		0.330		.08
	**Task 6**					
		Trial 1		0.097		.62
		Trial 10		0.412		.03

### Validity Hypothesis 3: Consequences of Testing

Among the reference group, statistically significant differences were found in the scores and the time taken to perform each task between the first and tenth trials. Among the experts, statistically significant differences were found in the scores in tasks 1 (*P*<.001), 3 (*P*=.03), and 4 (*P*=.01), and in the time taken to perform each task. These findings demonstrate a learning curve (Table 5).

**Table 5:** Score and time between trial 1 and trial 10, by level of training.

Metric and task		Comparison trials	Novices, *P* value	Experts, *P* value
**Score**				
	Task 1	1 vs 10	<.001	<.001
	Task 2	1 vs 10	.002	.052
	Task 3	1 vs 10	.002	.03
	Task 4	1 vs 10	<.001	.01
	Task 5	1 vs 10	<.001	.31
	Task 6	1 vs 10	.003	.63
**Time**				
	Task 1	1 vs 10	<.001	<.001
	Task 2	1 vs 10	<.001	<.001
	Task 3	1 vs 10	<.001	<.001
	Task 4	1 vs 10	<.001	<.001
	Task 5	1 vs 10	<.001	<.001
	Task 6	1 vs 10	<.001	<.001

In task 5, the reference group made statistically significantly fewer excess diathermy errors in the tenth trial than they did in the first trial (*P*=.003), which is evidence of a learning curve (Table 6).

**Table 6:** Excess diathermy errors when doing trials 1 and 10 in tasks 5 and 6, by level of training. Wilcoxon signed-rank test.

Task and group		Trial 1, mean (95% CI)	Trial 10, mean (95% CI)	*P* value
**Task 5**				
	Novices	0.205 (0.127, 0.282)	0.090 (0.031, 0.148)	.003
	Experts	0.125 (0.039, 0.210)	0.089 (0.013, 0.164)	.56
**Task 6**				
	Novices	0.200 (0.105, 0.294)	0.205 (0.120, 0.289)	.90
	Experts	0.250 (0.049, 0.450)	0.107 (0.010, 0.203)	.40

### Response Process Validity

Study participants had the opportunity to observe each task in advance by watching a video, and they received basic instruction. The only feedback the participants received was from the simulator; they did not receive any other type of feedback from the instructor. Each of the 177 tests performed (148 initial tests and 29 test-retests) was supervised by the same person (FAL). Photographic documentation of every person performing the tasks was obtained. The final performance scores were defined in advance by using the formula described in another study [53]. The exercise results were stored in an SQL database light within the simulator app itself after each test.

### SIMISGEST-VR Simulator Cost

The Leap Motion Controller costs approximately US $130, and the hardware elements cost approximately US $70. LapSim essence (Surgical Science Sweden AB) is a portable VR simulator that enables people to learn basic skills. It does not include haptics and is not available for sale, but it can be hired for 6 months at an approximate price of US $5500. To date, there are no publications about the validity of the tasks that this device proposes.

## Discussion

### General

The aim of this study was to evaluate a simulation instrument—SIMISGEST-VR—and to document the sources of validity evidence for task scores, relations to other variables, internal structure, consequences of testing, and response process.

Technology-enhanced simulation is defined as an “educational tool or device with which the learner physically interacts to mimic an aspect of clinical care for the purpose of teaching or assessment [83,84].” The use of simulators for learning basic psychomotor skills in minimally invasive surgery has been supported by multiple systematic reviews [16,17,85-90].

In the current state-of-the-art conceptual framework, validity is defined as the appropriate interpretation or use of test results and therefore applies only to the scores or interpretation in a specific context. The commonly used term *valid instrument* is inaccurate, and validity must be established for each intended interpretation [46,48,50]. Thus, when an evaluation instrument is said to be “valid” or to have been “validated,” it is essential to take into account the learning context, the performance context, the domain content, and the exigency of decisions taken on the basis of test results [91].

*Validation* refers to the process of collecting validity evidence to evaluate the appropriateness of the interpretations, uses, and decisions based on assessment results [52]. Validation is, therefore, a process and not an endpoint, and it involves gathering evidence and taking decisions based on the interpretation of the data obtained. In our case, validation required a series of experiments designed to provide evidence that the scores measured in SIMISGEST-VR reflected the technical skills they purported to measure [92].

The first step in any validity evaluation is to clearly define the construct. The construct we focused on validating was training and learning basic psychomotor skills in minimally invasive surgery using a low-cost portable simulator called SIMISGEST-VR. Several systematic reviews [16,93-96] have found that basic psychomotor skills can be learned in low-cost simulation models; however, low-cost simulators are often box trainers made from cardboard boxes [97], plastic crates [98], folding portable boxes [99], and boxes that require the use of laparoscopic equipment [100,101] or even an iPad [102]. There are no low-cost VR simulators on the market.

An important finding from this study was the high percentage of surgical residents and surgeons that had no experience with simulators, and the very low percentage of surgical residents who had experience with hybrid and VR simulators. This finding can be explained by the high cost of this type of simulator, which, in many countries, prevents the creation of simulation centers for learning basic psychomotor skills in minimally invasive surgery and constitutes an argument in favor of exploring the development of models of low-cost portable VR simulators such as SIMISGEST-VR. Ucelli [44] demonstrated a comparable outcome between supervised simulator practice and unstructured free simulator access without mentoring and, therefore, that “take home” simulation was both viable and economically beneficial.

### Validity Hypothesis 1: Relations to Other Variables

It is currently considered that a comparison between reference individuals and experts does not constitute an important validity argument [103,104]. However, it is the type of evidence for relations to other variables that is most often referred to in the literature [105]. The SIMISGEST-VR tasks were unable to demonstrate any difference in the performance scores between the reference group and the experts. A statistically significant difference was found between these 2 groups only in the time taken to complete tasks 3 (*P*=.006) and 6 (*P*=.02), which were the most complex.

Although some studies support the hypothesis that videogaming experience has a positive impact on minimally invasive surgery performance [106-113]. In this study, a significant difference was found for the reference group only in task 5 (diathermy; *P*=.003); in the other tasks, prior experience did not have any impact on performance. The demographic characterization made it clear that frequent videogaming (daily or weekly) was low in all population groups, which can explain the absence of impact on performance.

The lack of evidence for relations to other variables in this study can also be explained by the simplicity and ease of the proposed tasks.

### Validity Hypothesis 2: Internal Structure

The items demonstrated high internal consistency (Cronbach α=.81). A test should not be used if it has a Cronbach α<0.7, and it should not be used for important decisions on an individual unless the Cronbach α>0.9 [57,114-116]. In our case, therefore, the result enables us to support the use of SIMISGEST-VR tasks as a self-assessment test used for formative assessment [54].

Test-retest reliability is the correlation between scores for a test administered more than once among a homogeneous group of test takers at 2 different times (temporal stability); the longer the period of time, the less likely it is that a person will remember the simulator tasks and, therefore, the greater the test-retest threat will be [116-119]. In this study, the second exercise was performed 6 months after the first one, and the results obtained demonstrate significant evidence for the temporal stability of scores in the 6 tasks. When the metric used was time, similar results were obtained in all but tasks 4 (*P*=.048) and 6 (*P*=.03) when comparing the tenth trial.

### Validity Hypothesis 3: Evidence for Consequences of Testing

The most important finding of this study is that that the reference group learned in all the SIMISGEST-VR tasks. Excess diathermy error, defined as a contact time longer than 2 seconds from the moment of initial contact, fell significantly (*P*=.003) between the first and tenth trials for task 5 in the reference group, which also constitutes evidence for a learning curve. The experts group achieved a learning curve in all the tasks when time was taken as a metric, and for tasks 1 (*P*<.001), 3 (*P*=.03), and 4 (*P*=.01) when the final score of the test was taken into account. We, therefore, consider that the SIMISGEST-VR tasks can be used for the purpose of enabling novices without any prior experience to learn basic psychomotor skills in minimally invasive surgery.

This study has several strengths. The reference group sample included 100 students from 2 faculties of medicine, one public and one private; surgeons from a range of specialties; and surgical residents in general surgery and obstetric-gynecologic surgery. Physical simulators require the presence of a specialized tutor, a scarce, high-cost human resource, whereas VR simulators provide metrics and automatic feedback and allow the physical presence of a tutor to be dispensed with. At times of a pandemic such as COVID-19, this concept of education via VR takes on considerable significance because it avoids the need for learners to travel to simulation laboratories and, therefore, avoids close contact between students and instructors. This study also has limitations. Although the size of the reference group was large, a larger expert group would have been desirable. The sample size in our study was one of availability; as such, there are relatively more participants with minimal surgical experience compared to those with a lot of experience, such as senior surgical residents and surgeons. The low number of senior residents prevented significant results from being obtained when comparing them to the other groups. Another limitation of the data analysis in this study is that there was no statistical analysis performed before the trial to evaluate the proper sample size or to determine the Likert scale.

### Conclusions

This study has provided evidence to support the use of SIMISGEST-VR as a low-cost portable tool for the purpose of enabling novices without any prior experience to learn basic psychomotor skills in minimally invasive surgery. The tasks for learning basic motor skills in minimally invasive surgery demonstrated high internal consistency and high test-retest reliability among the reference group when assessing the task scores. The expert group also managed to obtain a learning curve in all the tasks when assessing the time metric. In this study, we were able to demonstrate partial evidence for relations to other variables and strong evidence for internal structure and test consequences.

Future work streams include the creation of different levels of difficulty in the tasks. We also intend to develop an app that can be downloaded online, which contains the full training program. Finally, we hope to develop simulation models using the Leap Motion Controller and other gesture-recognition devices such as the Myo armband.

## Appendix

Multimedia Appendix 1 Form for the demographic questionnaire.

## Abbreviations

MIST-VR: Minimally Invasive Surgery Training - Virtual Reality
SIMISGEST-VR: Simulator of Minimally Invasive Surgery mediated by Gestures - Virtual Reality
VR: virtual reality

## References

[1] Litynski GS (1999). Profiles in laparoscopy: Mouret, Dubois, and Perissat: the laparoscopic breakthrough in Europe (1987-1988). JSLS.

[2] Yamashita Y, Kurohiji T, Kakegawa T (1994). Evaluation of two training programs for laparoscopic cholecystectomy: incidence of major complications. World J Surg.

[3] Archer SB, Brown DW, Smith CD, Branum GD, Hunter JG (2001). Bile duct injury during laparoscopic cholecystectomy: results of a national survey. Ann Surg.

[4] Berci G (1998). Complications of laparoscopic cholecystectomy. Surg Endosc.

[5] Rogers DA, Elstein AS, Bordage G (2001). Improving continuing medical education for surgical techniques: applying the lessons learned in the first decade of minimal access surgery. Ann Surg.

[6] Hugh TB (2002). New strategies to prevent laparoscopic bile duct injury--surgeons can learn from pilots. Surgery.

[7] MacFadyen BV, Vecchio R, Ricardo AE, Mathis CR (1998). Bile duct injury after laparoscopic cholecystectomy. the United States experience. Surg Endosc.

[8] Wolfe BM, Gardiner B, Frey CF (2015). Laparoscopic cholecystectomy: a remarkable development. JAMA.

[9] Seymour NE, Gallagher AG, Roman SA, O'Brien Michael K, Bansal VK, Andersen DK, Satava RM (2002). Virtual reality training improves operating room performance: results of a randomized, double-blinded study. Ann Surg.

[10] Gallagher AG, Richie K, McClure N, McGuigan J (2001). Objective psychomotor skills assessment of experienced, junior, and novice laparoscopists with virtual reality. World J Surg.

[11] Gallagher AG, Smith CD, Bowers SP, Seymour NE, Pearson A, McNatt S, Hananel D, Satava RM (2003). Psychomotor skills assessment in practicing surgeons experienced in performing advanced laparoscopic procedures. J Am Coll Surg.

[12] Korndorffer JR, Dunne JB, Sierra R, Stefanidis D, Touchard CL, Scott DJ (2005). Simulator training for laparoscopic suturing using performance goals translates to the operating room. J Am Coll Surg.

[13] Aggarwal R, Ward J, Balasundaram I, Sains P, Athanasiou T, Darzi A (2007). Proving the effectiveness of virtual reality simulation for training in laparoscopic surgery. Ann Surg.

[14] Sturm Lana P, Windsor John A, Cosman PH, Cregan P, Hewett PJ, Maddern GJ (2008). A systematic review of skills transfer after surgical simulation training. Ann Surg.

[15] Seymour NE (2008). VR to OR: a review of the evidence that virtual reality simulation improves operating room performance. World J Surg.

[16] Zendejas B, Brydges R, Hamstra SJ, Cook DA (2013). State of the evidence on simulation-based training for laparoscopic surgery: a systematic review. Ann Surg.

[17] Dawe SR, Pena GN, Windsor JA, Broeders JAJL, Cregan PC, Hewett PJ, Maddern GJ (2014). Systematic review of skills transfer after surgical simulation-based training. Br J Surg.

[18] Hyltander A, Liljegren E, Rhodin P, Lönroth H (2002). The transfer of basic skills learned in a laparoscopic simulator to the operating room. Surg Endosc.

[19] Youngblood PL, Srivastava S, Curet M, Heinrichs WL, Dev P, Wren SM (2005). Comparison of training on two laparoscopic simulators and assessment of skills transfer to surgical performance. J Am Coll Surg.

[20] Sutton C, McCloy R, Middlebrook A, Chater P, Wilson M, Stone R (1997). MIST VR. A laparoscopic surgery procedures trainer and evaluator. Stud Health Technol Inform.

[21] Ahlberg G, Heikkinen T, Iselius L, Leijonmarck C, Rutqvist J, Arvidsson D (2002). Does training in a virtual reality simulator improve surgical performance?. Surg Endosc.

[22] Debes AJ, Aggarwal R, Balasundaram I, Jacobsen MB (2010). A tale of two trainers: virtual reality versus a video trainer for acquisition of basic laparoscopic skills. Am J Surg.

[23] Kothari SN, Kaplan BJ, DeMaria EJ, Broderick TJ, Merrell RC (2002). Training in laparoscopic suturing skills using a new computer-based virtual reality simulator (MIST-VR) provides results comparable to those with an established pelvic trainer system. J Laparoendosc Adv Surg Tech A.

[24] Torkington J, Smith S, Rees B, Darzi A (2001). Skill transfer from virtual reality to a real laparoscopic task. Surg Endosc.

[25] Torkington J, Smith S, Rees B, Darzi A (2001). The role of the basic surgical skills course in the acquisition and retention of laparoscopic skill. Surg Endosc.

[26] Alvarez-Lopez F, Maina MF, Saigí-Rubió Francesc (2019). Use of commercial off-the-shelf devices for the detection of manual gestures in surgery: systematic literature review. J Med Internet Res.

[27] Ogura T, Sato M, Ishida Y, Hayashi N, Doi K (2014). Development of a novel method for manipulation of angiographic images by use of a motion sensor in operating rooms. Radiol Phys Technol.

[28] Mauser S, Burgert O (2014). Touch-free, gesture-based control of medical devices and software based on the leap motion controller. Stud Health Technol Inform.

[29] Bachmann D, Weichert F, Rinkenauer G (2014). Evaluation of the leap motion controller as a new contact-free pointing device. Sensors (Basel).

[30] Weichert F, Bachmann D, Rudak B, Fisseler D (2013). Analysis of the accuracy and robustness of the leap motion controller. Sensors (Basel).

[31] Guna J, Jakus G, Pogačnik M, Tomažič S, Sodnik J (2014). An analysis of the precision and reliability of the leap motion sensor and its suitability for static and dynamic tracking. Sensors (Basel).

[32] Alvarez-Lopez F, Maina MF, Saigí-Rubió Francesc (2016). Natural User Interfaces: Is It a Solution to Accomplish Ubiquitous Training in Minimally Invasive Surgery?. Surg Innov.

[33] Oropesa I, de Jong T, Sánchez-González P, Dankelman J, Gómez E (2016). Feasibility of tracking laparoscopic instruments in a box trainer using a Leap Motion Controller. Measurement.

[34] Beck P (2016). Accurate three-dimensional instrument positioning. Free Patents Online.

[35] Lahanas V, Loukas C, Georgiou K, Lababidi H, Al-Jaroudi D (2017). Virtual reality-based assessment of basic laparoscopic skills using the Leap Motion controller. Surg Endosc.

[36] Travaglini T, Swaney P, Weaver K, Webster R (2016). Initial experiments with the leap motion as a user interface in robotic endonasal surgery. Robot Mechatron (2015).

[37] Juanes JA, Gómez Juan J, Peguero PD, Ruisoto P (2016). Digital environment for movement control in surgical skill training. J Med Syst.

[38] Partridge RW, Brown FS, Brennan PM, Hennessey IAM, Hughes MA (2016). The LEAPTM gesture interface device and take-home laparoscopic simulators: a study of construct and concurrent validity. Surg Innov.

[39] Wright T, de Ribaupierre S, Eagleson R (2017). Design and evaluation of an augmented reality simulator using leap motion. Healthc Technol Lett.

[40] Botden SMBI, Jakimowicz JJ (2009). What is going on in augmented reality simulation in laparoscopic surgery?. Surg Endosc.

[41] Papanikolaou IG (2013). Assessment of medical simulators as a training programme for current surgical education. Hellenic J Surg.

[42] Hennessey IA, Hewett P (2013). Construct, concurrent, and content validity of the eoSim laparoscopic simulator. J Laparoendosc Adv Surg Tech A.

[43] Hruby GW, Sprenkle PC, Abdelshehid C, Clayman RV, McDougall EM, Landman J (2008). The EZ Trainer: validation of a portable and inexpensive simulator for training basic laparoscopic skills. J Urol.

[44] Uccelli J, Kahol K, Ashby A, Smith M, Ferrara J (2011). The validity of take-home surgical simulators to enhance resident technical skill proficiency. Am J Surg.

[45] (2018). Estándares Para Pruebas Educativas y Psicológicas. JSTOR.

[46] American Educational Research Association, American Psychological Association, National Council on Measurement in Education (2014). Standards for Educational and Psychological Testing.

[47] Downing SM (2003). Validity: on meaningful interpretation of assessment data. Med Educ.

[48] Cook DA, Beckman TJ (2006). Current concepts in validity and reliability for psychometric instruments: theory and application. Am J Med.

[49] Cook DA, Zendejas B, Hamstra SJ, Hatala R, Brydges R (2014). What counts as validity evidence? Examples and prevalence in a systematic review of simulation-based assessment. Adv Health Sci Educ Theory Pract.

[50] Messick S (1995). Validity of psychological assessment: Validation of inferences from persons' responses and performances as scientific inquiry into score meaning. American Psychologist.

[51] Messick S (2016). Meaning and values in test validation: the science and ethics of assessment. Educational Researcher.

[52] Cook DA, Hatala R (2016). Validation of educational assessments: a primer for simulation and beyond. Adv Simul (Lond).

[53] Alvarez-Lopez F, Maina MF, Saigí-Rubió Francesc (2020). Use of a low-cost portable 3d virtual reality gesture-mediated simulator for training and learning basic psychomotor skills in minimally invasive surgery: development and content validity study. J Med Internet Res.

[54] Ghaderi I, Manji F, Park Yoon Soo, Juul D, Ott M, Harris I, Farrell Tm (2015). Technical skills assessment toolbox: a review using the unitary framework of validity. Ann Surg.

[55] Beckman TJ, Cook DA, Mandrekar JN (2005). What is the validity evidence for assessments of clinical teaching?. J Gen Intern Med.

[56] Sweet Rm, Hananel D, Lawrenz F (2010). A unified approach to validation, reliability, and education study design for surgical technical skills training. Arch Surg.

[57] Gallagher AG, Ritter EM, Satava RM (2003). Fundamental principles of validation, and reliability: rigorous science for the assessment of surgical education and training. Surg Endosc.

[58] Worster A, Haines T (2004). Advanced statistics: understanding medical record review (MRR) studies. Acad Emerg Med.

[59] Warner B (2017). The sciences of the artificial. Journal of the Operational Research Society.

[60] Manson N (2006). Is operations research really research?. ORiON.

[61] Dresch A, Pacheco-Lacerda D, Valle-Antunes J (2015). Design science research. A Method for Science and Technology Advancement.

[62] Lacerda DP, Dresch A, Proença A, Antunes Júnior JAV (2013). Design Science Research: método de pesquisa para a engenharia de produção. Gest. Prod.

[63] Hevner A, Chatterjee S (2010). Design research in information systems. Theory and Practice.

[64] Wilson Ms, Middlebrook A, Sutton C, Stone R, McCloy Rf (1997). MIST VR: a virtual reality trainer for laparoscopic surgery assesses performance. Ann R Coll Surg Engl.

[65] Ali M, Mowery Y, Kaplan B, DeMaria E (2002). Training the novice in laparoscopy. More challenge is better. Surg Endosc.

[66] Grantcharov TP, Rosenberg J, Pahle E, Funch-Jensen P (2001). Virtual reality computer simulation. Surg Endosc.

[67] Hamilton E, Scott D, Fleming J, Rege R, Laycock R, Bergen P, Tesfay S, Jones D (2002). Comparison of video trainer and virtual reality training systems on acquisition of laparoscopic skills. Surg Endosc.

[68] Grantcharov TP, Kristiansen VB, Bendix J, Bardram L, Rosenberg J, Funch-Jensen P (2004). Randomized clinical trial of virtual reality simulation for laparoscopic skills training. Br J Surg.

[69] Gonzalez R, Bowers SP, Smith CD, Ramshaw BJ (2004). Does setting specific goals and providing feedback during training result in better acquisition of laparoscopic skills?. Am Surg.

[70] McClusky DA, Gallagher AG, Ritter E, Lederman AB, Van Sickle KR, Baghai M, Smith C (2004). Virtual reality training improves junior residents’ operating room performance: Results of a prospective, randomized, double-blinded study of the complete laparoscopic cholecystectomy. Journal of the American College of Surgeons.

[71] Grantcharov TP, Funch-Jensen P (2009). Can everyone achieve proficiency with the laparoscopic technique? Learning curve patterns in technical skills acquisition. Am J Surg.

[72] Gallagher AG, Seymour NE, Jordan-Black J, Bunting BP, McGlade K, Satava RM (2013). Prospective, Randomized Assessment of Transfer of Training (ToT) and Transfer Effectiveness Ratio (TER) of Virtual Reality Simulation Training for Laparoscopic Skill Acquisition. Annals of Surgery.

[73] Badash I, Burtt K, Solorzano CA, Carey JN (2016). Innovations in surgery simulation: a review of past, current and future techniques. Ann Transl Med.

[74] Taffinder N, Sutton C, Fishwick RJ, McManus IC, Darzi A (1998). Validation of virtual reality to teach and assess psychomotor skills in laparoscopic surgery: results from randomised controlled studies using the MIST VR laparoscopic simulator. Stud Health Technol Inform.

[75] Chaudhry A, Sutton C, Wood J, Stone R, McCloy R (1999). Learning rate for laparoscopic surgical skills on MIST VR, a virtual reality simulator: quality of human-computer interface. Ann R Coll Surg Engl.

[76] Stylopoulos N, Vosburgh KG (2007). Assessing technical skill in surgery and endoscopy: a set of metrics and an algorithm (C-PASS) to assess skills in surgical and endoscopic procedures. Surg Innov.

[77] Hatala R, Cook DA, Zendejas B, Hamstra SJ, Brydges R (2014). Feedback for simulation-based procedural skills training: a meta-analysis and critical narrative synthesis. Adv Health Sci Educ Theory Pract.

[78] Gallagher A, Satava R (2002). Virtual reality as a metric for the assessment of laparoscopic psychomotor skills. Learning curves and reliability measures. Surg Endosc.

[79] Archer J (2010). State of the science in health professional education: effective feedback. Med Educ.

[80] Walsh CM, Ling SC, Wang CS, Carnahan H (2009). Concurrent versus terminal feedback: it may be better to wait. Academic Medicine.

[81] Grantcharov TP, Schulze S, Kristiansen VB (2007). The impact of objective assessment and constructive feedback on improvement of laparoscopic performance in the operating room. Surg Endosc.

[82] Stefanidis D, Korndorffer JR, Heniford BT, Scott DJ (2007). Limited feedback and video tutorials optimize learning and resource utilization during laparoscopic simulator training. Surgery.

[83] Cook DA, Brydges R, Zendejas B, Hamstra SJ, Hatala R (2013). Technology-enhanced simulation to assess health professionals: a systematic review of validity evidence, research methods, and reporting quality. Acad Med.

[84] Cook DA, Hatala R, Brydges R, Zendejas B, Szostek JH, Wang AT, Erwin PJ, Hamstra SJ (2011). Technology-enhanced simulation for health professions education: a systematic review and meta-analysis. JAMA.

[85] Haque S, Srinivasan S (2006). A meta-analysis of the training effectiveness of virtual reality surgical simulators. IEEE Trans Inf Technol Biomed.

[86] Brydges R, Hatala R, Zendejas B, Erwin PJ, Cook DA (2015). Linking simulation-based educational assessments and patient-related outcomes: a systematic review and meta-analysis. Acad Med.

[87] Nagendran M, Gurusamy KS, Aggarwal R, Loizidou M, Davidson BR (2013). Virtual reality training for surgical trainees in laparoscopic surgery. Cochrane Database Syst Rev.

[88] Thomas MP (2013). The role of simulation in the development of technical competence during surgical training: a literature review. Int J Med Educ.

[89] Dawe SR, Windsor JA, Broeders JA, Cregan PC, Hewett PJ, Maddern GJ (2014). A systematic review of surgical skills transfer after simulation-based training: laparoscopic cholecystectomy and endoscopy. Ann Surg.

[90] Gurusamy K, Aggarwal R, Palanivelu L, Davidson BR (2008). Systematic review of randomized controlled trials on the effectiveness of virtual reality training for laparoscopic surgery. Br J Surg.

[91] Andreatta P, Gruppen L (2009). Conceptualising and classifying validity evidence for simulation. Med Educ.

[92] Fried GM, Feldman LS, Vassiliou MC, Fraser SA, Stanbridge D, Ghitulescu G, Andrew CG (2004). Proving the value of simulation in laparoscopic surgery. Ann Surg.

[93] Li MM, George J (2017). A systematic review of low-cost laparoscopic simulators. Surg Endosc.

[94] Nguyen T, Braga LH, Hoogenes J, Matsumoto ED (2013). Commercial video laparoscopic trainers versus less expensive, simple laparoscopic trainers: a systematic review and meta-analysis. J Urol.

[95] Nagendran M, Toon C, Davidson B, Gurusamy K (2014). Laparoscopic surgical box model training for surgical trainees with no prior laparoscopic experience. Cochrane Database Syst Rev.

[96] Diesen DL, Erhunmwunsee L, Bennett KM, Ben-David K, Yurcisin B, Ceppa EP, Omotosho PA, Perez A, Pryor A (2011). Effectiveness of laparoscopic computer simulator versus usage of box trainer for endoscopic surgery training of novices. J Surg Educ.

[97] Blacker AJ (2005). How to build your own laparoscopic trainer. J Endourol.

[98] Smith MD, Norris JM, Kishikova L, Smith DP (2013). Laparoscopic simulation for all: two affordable, upgradable, and easy-to-build laparoscopic trainers. J Surg Educ.

[99] Nakamura LY, Martin GL, Fox JC, Andrews PE, Humphreys M, Castle EP (2012). Comparing the portable laparoscopic trainer with a standardized trainer in surgically naïve subjects. J Endourol.

[100] Ricchiuti D, Ralat DA, Evancho-Chapman M, Wyneski H, Cerone J, Wegryn JD (2005). A simple cost-effective design for construction of a laparoscopic trainer. J Endourol.

[101] Mughal M (1992). A cheap laparoscopic surgery trainer. Ann R Coll Surg Engl.

[102] Ruparel RK, Brahmbhatt RD, Dove JC, Hutchinson RC, Stauffer JA, Bowers SP, Richie E, Lannen AM, Thiel DD (2014). "iTrainers"--novel and inexpensive alternatives to traditional laparoscopic box trainers. Urology.

[103] Cook DA (2015). Much ado about differences: why expert-novice comparisons add little to the validity argument. Adv Health Sci Educ Theory Pract.

[104] Zendejas B, Ruparel RK, Cook DA (2016). Validity evidence for the Fundamentals of Laparoscopic Surgery (FLS) program as an assessment tool: a systematic review. Surg Endosc.

[105] Borgersen NJ, Naur TMH, Sørensen Stine M D, Bjerrum F, Konge L, Subhi Y, Thomsen ASS (2018). Gathering validity evidence for surgical simulation: a systematic review. Ann Surg.

[106] Rosser JC, Lynch Paul J, Cuddihy Laurie, Gentile Douglas A, Klonsky Jonathan, Merrell Ronald (2007). The impact of video games on training surgeons in the 21st century. Arch Surg.

[107] van Dongen KW, Verleisdonk EMM, Schijven MP, Broeders IAMJ (2011). Will the Playstation generation become better endoscopic surgeons?. Surg Endosc.

[108] Grantcharov TP, Bardram L, Funch-Jensen P, Rosenberg J (2003). Impact of hand dominance, gender, and experience with computer games on performance in virtual reality laparoscopy. Surg Endosc.

[109] Boyle E, Kennedy A, Traynor O, Hill ADK (2011). Training surgical skills using nonsurgical tasks--can Nintendo Wii™ improve surgical performance?. J Surg Educ.

[110] Harper JD, Kaiser S, Ebrahimi K, Lamberton GR, Hadley HR, Ruckle HC, Baldwin DD (2007). Prior video game exposure does not enhance robotic surgical performance. J Endourol.

[111] Glaser AY, Hall CB, Uribe JI, Fried MP (2005). The effects of previously acquired skills on sinus surgery simulator performance. Otolaryngol Head Neck Surg.

[112] Madan AK, Frantzides CT, Park WC, Tebbit CL, Kumari NVA, O'Leary PJ (2005). Predicting baseline laparoscopic surgery skills. Surg Endosc.

[113] Kennedy A, Boyle E, Traynor O, Walsh T, Hill A (2011). Video gaming enhances psychomotor skills but not visuospatial and perceptual abilities in surgical trainees. J Surg Educ.

[114] Wanzel KR, Ward M, Reznick RK (2002). Teaching the surgical craft: from selection to certification. Curr Probl Surg.

[115] Cronbach LJ, Meehl PE (1955). Construct validity in psychological tests. Psychological Bulletin.

[116] Feldman LS, Sherman V, Fried GM (2004). Using simulators to assess laparoscopic competence: ready for widespread use?. Surgery.

[117] Straub D, Gefen D (2004). Validation Guidelines for IS Positivist Research. CAIS.

[118] McDougall EM (2007). Validation of surgical simulators. J Endourol.

[119] Kowalewski K, Hendrie JD, Schmidt MW, Garrow CR, Bruckner T, Proctor T, Paul S, Adigüzel Davud, Bodenstedt S, Erben A, Kenngott H, Erben Y, Speidel S, Müller-Stich Beat P, Nickel F (2017). Development and validation of a sensor- and expert model-based training system for laparoscopic surgery: the iSurgeon. Surg Endosc.

