# Supplementary material for: Use of a Low-Cost Portable 3D Virtual Reality Simulator for Psychomotor Skill Training in Minimally Invasive Surgery: Task Metrics and Score Validity
Source: JMIR Serious Games. 2020 Oct 27;8(4):e19723. doi: 10.2196/19723 (PMC7655469; doi:10.2196/19723)
Supplement: Multimedia Appendix 1 [file games_v8i4e19723_app1.docx]

**Appendix.**

**Appendix 1.** Form for the demographic questionnaire.

- 1. Demographic questionnaire

Name ______________________________________________________

Age______

Gender ______

Nationality _______________

Dominant hand: Right___ Left___ Ambidextrous___

Do you have prior experience with simulators in minimally invasive surgery? **YES __ NO __**

If YES, what type of simulator?

- Physical___
- Hybrid/augmented virtual reality ___
- Virtual reality ___

Do you have regular videogaming experience? **YES __ NO __**

If YES, how often do you play video games?

| Daily | Weekly | Monthly | Occasionally |
| --- | --- | --- | --- |

Have you had previous experience with virtual reality devices **YES ___ NO ___**

If YES, how often do you perform activities with virtual/augmented reality devices?

| Daily | Weekly | Monthly | Occasionally |
| --- | --- | --- | --- |

Select the most appropriate answer for your level of training

1. Undergraduate student
2. Surgical resident
3. Practicing surgeon
4. Other. Please specify ___________________

Select which of these options describes your level of experience in laparoscopic surgery:

1. None
2. Basic camera manipulation and/or forceps retraction
3. Basic operating level (cholecystectomy, appendectomy)
4. Intermediate operating level (fundoplication)
5. Advanced level
